# Synchronous Multifocal Necrotizing Soft Tissue Infection of the Upper and Lower Limbs in a Patient With Diabetic Ketoacidosis: A Report of a Rare Case

**DOI:** 10.7759/cureus.101194

**Published:** 2026-01-09

**Authors:** Tetsushi Nasu, Eri Tamagawa, Atsuyo Fujita, Akiko Hashimoto, Ritsuko Kimura

**Affiliations:** 1 Diabetes Medicine, Hannan Municipal Hospital, Hannan, JPN; 2 Internal Medicine, Hannan Municipal Hospital, Hannan, JPN

**Keywords:** diabetes, diabetic ketoacidosis (dka), multifocal, necrotizing fasciitis, necrotizing soft tissue infection

## Abstract

A 67-year-old man with no history of diabetes presented with generalized body pain and impaired mobility. He was admitted with severe hyperglycemia, diabetic ketoacidosis, and septic shock. Although the patient experienced minimal cutaneous symptoms, he experienced progressive swelling and pain in the right upper and left lower extremities. Whole-body imaging revealed an extensive fascial plane abscess. He underwent three surgical debridements and improved with prolonged antibiotic therapy. Necrotizing soft tissue infections are rapidly progressing and life-threatening. This case highlights a rare form of hematogenous spread that causes simultaneous multifocal soft-tissue infections rather than extending from a single localized source.

## Introduction

Descriptions of necrotizing soft-tissue infections (NSTI) can be traced back to Hippocrates in the fifth century BC, though formal clinical descriptions are generally recognized to date from the late 18th century [1]. The term “Fournier’s gangrene” is used to describe necrotizing infections of the perineum and scrotum, illustrating how the nomenclature often varies according to the anatomical site involved. Since Wilson first introduced the term “necrotizing fasciitis (NF)” in the 1950s, several overlapping terms have been used to describe similar diseases [2,3]. Because the infection process involves not only the fascia but also other soft tissues, including the skin, subcutaneous tissue, and muscle, the broader term “NSTI” has recently been adopted in the clinical literature. This diversity of terminology reflects historical evolution but also contributes to inconsistency in classification and reporting across studies. In this report, the term “NSTI” is used as an inclusive designation that encompasses NF. Although several previous studies have referred to this condition as NF, we adopted NSTI as the unified term for clarity and consistency throughout this study [4].

The incidence of NSTI is estimated to be 0.3-5.0 per 100,000 individuals annually, which is markedly lower than that of cellulitis [4-8]. NSTI develops following minor trauma, burns, or other cutaneous insults and progresses to disseminated intravascular coagulation (DIC) and sepsis. The overall mortality rate of NSTI remains high at approximately 20-70% [8-10]. Compared to monofocal necrotizing soft tissue infection (MoNSTI), which develops locally, synchronous multifocal necrotizing soft tissue infection (SMNSTI) is even rarer and carries a higher risk of severe disease, with a mortality rate of up to 68.4% [11]. Considering its fulminant clinical course, limb amputation is frequently required, and early diagnosis with prompt surgical and medical intervention remains a key strategy for limiting disease progression. Appropriate antibiotic therapy and hemodynamic support play essential roles in influencing clinical outcomes.

Here, we describe a rare case of an SMNSTI in the upper and lower extremities associated with diabetic ketoacidosis (DKA), which was successfully managed without amputation.

## Case presentation

A 67-year-old man presented to our hospital with generalized pain and progressive difficulty in ambulation and limb movement. He reported an onset of urinary frequency three weeks before admission. One week before the presentation, he had developed diffuse pain, predominantly involving the extremities, which progressively worsened and rendered him unable to ambulate without family assistance. The patient had no relevant medical history.

On admission, the patient became alert and oriented. His vital signs revealed a body temperature of 36.2°C, and he was afebrile. His blood pressure was 94/63 mmHg, pulse rate was 113 beats/min, and oxygen saturation was 97% in room air. These findings were consistent with mild hypotension and tachycardia. Physical examination revealed a dry oral mucosa. No abnormal findings were noted on chest or abdominal examinations. Mild edema was observed in the extremities. The right upper extremity could not be elevated because of severe pain. In contrast, the left upper extremity and both lower extremities could be elevated but were limited by significant pain, causing impaired mobility. No cutaneous manifestations, such as blisters, erythema, or traumatic injuries, were found. The laboratory findings on admission are summarized in Table 1.

**Table 1:** Laboratory findings on admission Reference ranges are based on the standards of our institutional clinical laboratory.

Parameter	Result	Reference range
Complete blood count		
Hemoglobin	15.4 g/dL	13.7-16.8 g/dL
White blood cells	27.0 × 10⁹/L	3.3-8.6 × 10⁹/L
Platelets	299 × 10⁹/L	158-348 × 10⁹/L
Blood chemistry		
Sodium	117 mmol/L	138-145 mmol/L
Potassium	4.1 mmol/L	3.6-4.8 mmol/L
Chloride	79 mmol/L	101-108 mmol/L
Blood urea nitrogen	61.5 mg/dL	8.0-20.0 mg/dL
Creatinine	2.25 mg/dL	0.65-1.07 mg/dL
Aspartate aminotransferase	47 U/L	13-30 U/L
Alanine aminotransferase	37 U/L	10-42 U/L
Glucose	654 mg/dL	73-109 mg/dL
Glycated hemoglobin	11.60%	4.9-6.0%
C-reactive protein	36.77 mg/dL	<0.14 mg/dL
Procalcitonin	67.95 ng/mL	<0.50 ng/dL
Coagulation profile		
Prothrombin time	14.6 seconds	9.6-13.1 seconds
International normalized ratio	1.42	0.8-1.2
Activated partial thromboplastin time	35.4 seconds	24.0-34.0 seconds
Fibrinogen	>900 mg/dL	170.0-400.0 mg/dL
D-dimer	18.6 μ/L	<1.0 μ/L
Urinalysis		
Appearance	Amber	
Protein	2+	Negative
Glucose	2+	Negative
Red blood cells	20-29/HPF	<5/HPF
White blood cells	>100/HPF	<5/HPF
Ketone	+/-	Negative
Venous blood gas (room air)		
pH	7.274	7.32-7.43
PvCO₂	34.6 mmHg	38.0-50.0 mmHg
HCO₃	15.8 mmol/L	22.0-26.0 mmol/L
Base excess	-10.1 mmol/L	-2.0 to +2.0 mmol/L

The patient exhibited marked leukocytosis, with significantly elevated C-reactive protein and procalcitonin levels. Hyperglycemia and elevated glycated hemoglobin levels suggested previously undiagnosed, poorly controlled diabetes in the setting of severe infection. Furthermore, venous blood gas analysis revealed metabolic acidosis, consistent with concomitant DKA. The endogenous insulin secretion, as measured by C-peptide levels, was 12.0 ng/mL (reference range: 0.61-2.09 ng/mL). This demonstrated preserved insulin production and ruled out insulin deficiency, making type 1 diabetes unlikely. The source of infection was not apparent on initial evaluation, and the patient was admitted to a high-care unit (intermediate intensive care unit) for intensive management.

Insulin infusion and fluid resuscitation with normal saline were initiated promptly. As the patient developed septic shock, continuous norepinephrine was administered via a central venous catheter. Carbapenem-class antibiotics were concurrently administered.

On the third day of admission, lower extremity venous ultrasonography was performed to screen for deep vein thrombosis (DVT). The scan revealed thrombotic occlusions with vascular dilation in the right gastrocnemius vein and the left soleal vein. These findings were consistent with sepsis-associated DVT, and anticoagulation therapy with heparin was initiated.

**Figure 1:**
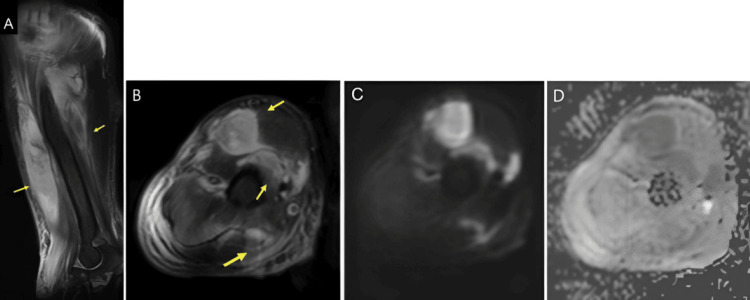
MRI of the right upper limb On day 8 after admission, intramuscular lesions with high signal intensity in the right branchial biceps muscle and brachial triceps muscle are depicted on sagittal and axial fat suppression T2WI MRI (A, B) and DWI (C), and ADC values are relatively low (0.95-1.403) (D), indicative of abscess formation. ADC, apparent diffusion coefficient; DWI, diffusion-weighted imaging; T2WI, T2-weighted imaging

The patient initially demonstrated improvement in septic shock and DKA, with stabilization of the respiratory and circulatory dynamics under treatment. However, the pain in the right upper limb persisted after admission and was gradually accompanied by swelling, erythema, and warmth of the overlying skin. Based on the Laboratory Risk Indicator for Necrotizing Fasciitis score [12] of 10 points (reference range: <6 points), an NSTI of the right upper limb was strongly suspected. MRI of the right upper limb revealed an intramuscular abscess (Figure 1).

In response to the clinical course, initial empirical therapy was adjusted to intravenous cefazolin sodium hydrate and clindamycin. However, surgical debridement of the right upper limb was performed due to the lack of clinical improvement on hospital day 14.

**Figure 2:**
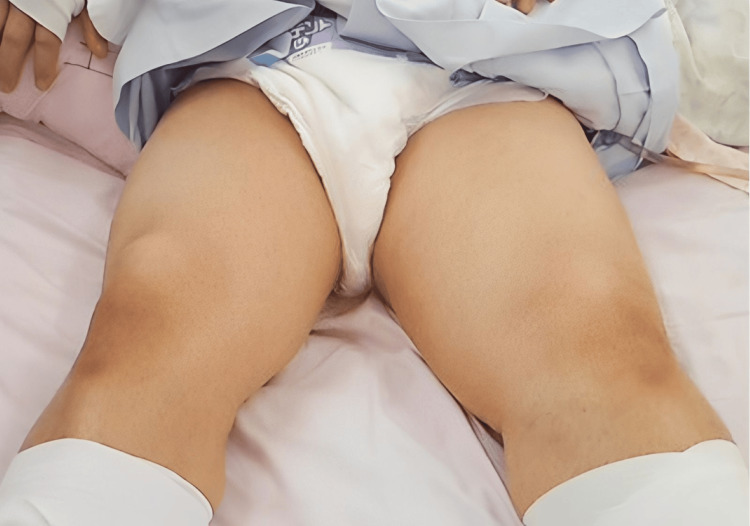
Gross appearance of both thighs on day 14 after admission The maximum circumference of the left thigh was 59 cm, in contrast to 55 cm of the right thigh, indicating marked swelling on the left side. Erythema and purpura were not observed on the overlying skin.

The patient simultaneously developed pain and swelling in the left lower extremity. The maximum circumference of the left thigh was 59 cm, compared with 55 cm in the right thigh, indicating apparent asymmetry (Figure 2).

**Figure 3:**
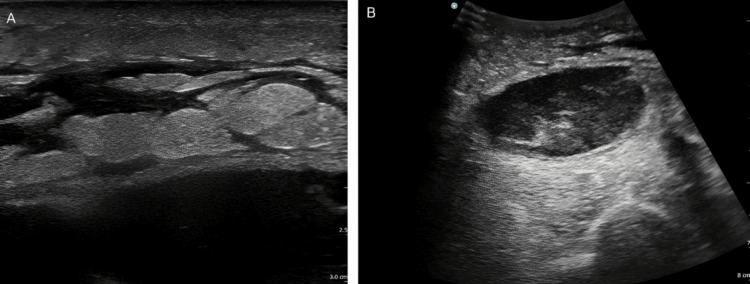
Superficial ultrasound image of the left thigh A high-frequency linear probe (3-12 MHz) was used for superficial ultrasonography, which revealed a cobblestone appearance in the subcutaneous tissue (A). Deep ultrasonography with a low-frequency convex probe (2-5 MHz) demonstrated a mixed hyperechoic and hypoechoic fluid collection along the deep fascial layer, suggestive of abscess formation (B).

Although the cutaneous findings on the lateral aspect of the left thigh were minimal, ultrasonography was performed because multiple deep-seated soft tissue infections were suspected. Superficial ultrasonography (Figure 3A) revealed a cobblestone appearance in the subcutaneous adipose tissue. By contrast, deeper imaging (Figure 3B) revealed mixed hyperechoic and hypoechoic fluid collections within the deep fascial layers, suggesting abscess formation.

**Figure 4:**
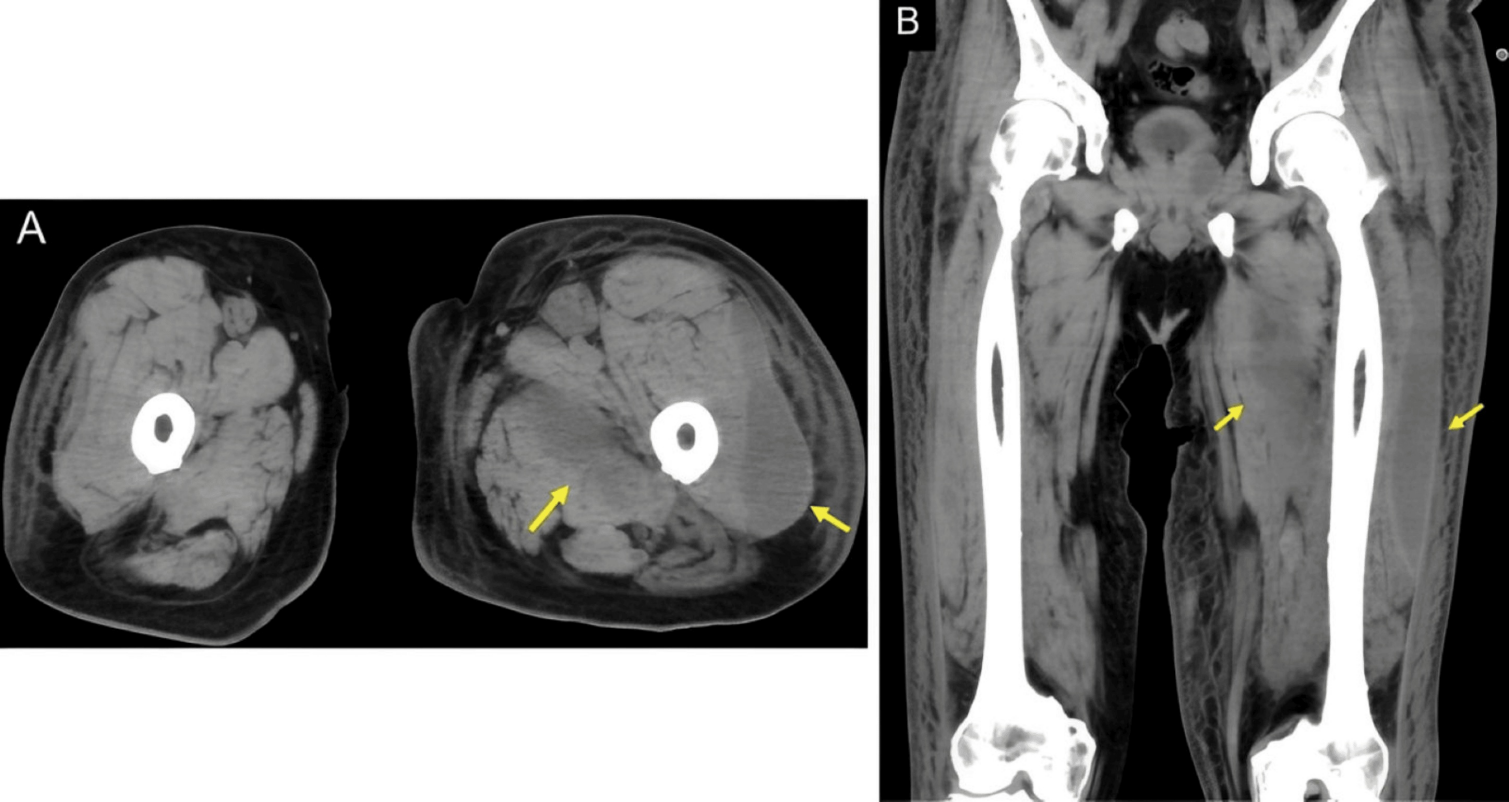
CT image of the thigh On day 17 after admission, axial (A) and coronal (B) CT images reveal a swollen left thigh with intramuscular fluid retention within the vastus lateralis (arrow) and adductor brevis muscles.

**Figure 5:**
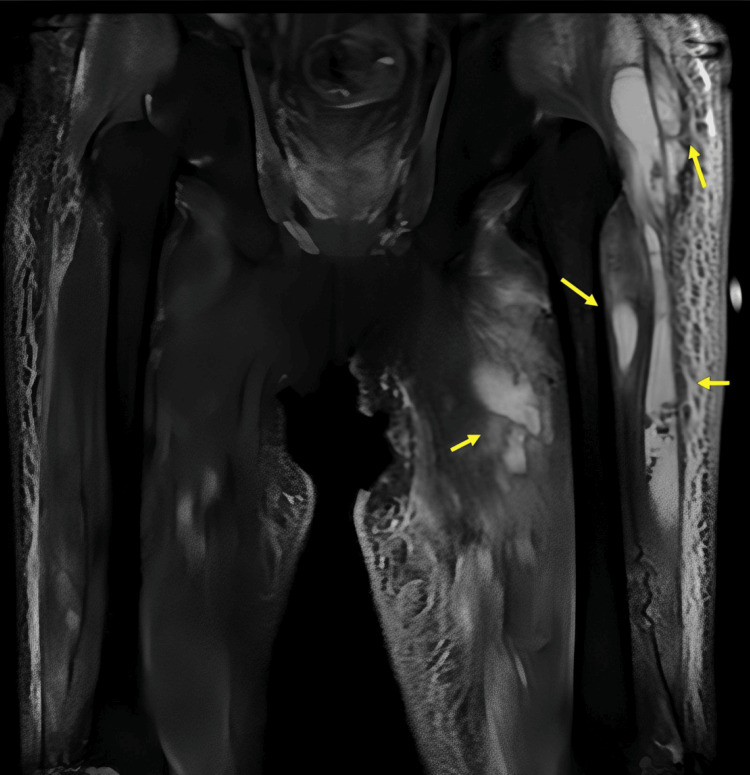
MRI of the thigh On day 21, intramuscular fluid retention with high signal intensity involving the gluteus medius, vastus lateralis, vastus medialis, and adductor brevis muscles is depicted (arrow) on coronal fat-suppressed T2WI. Note that low-signal-intensity spots corresponding to air bubbles are present in the fluid of the vastus lateralis muscle, consistent with an abscess. T2WI, T2-weighted imaging

Subsequent CT and MRI of the left thigh confirmed intramuscular abscesses (Figure 4, Figure 5).

**Figure 6:**
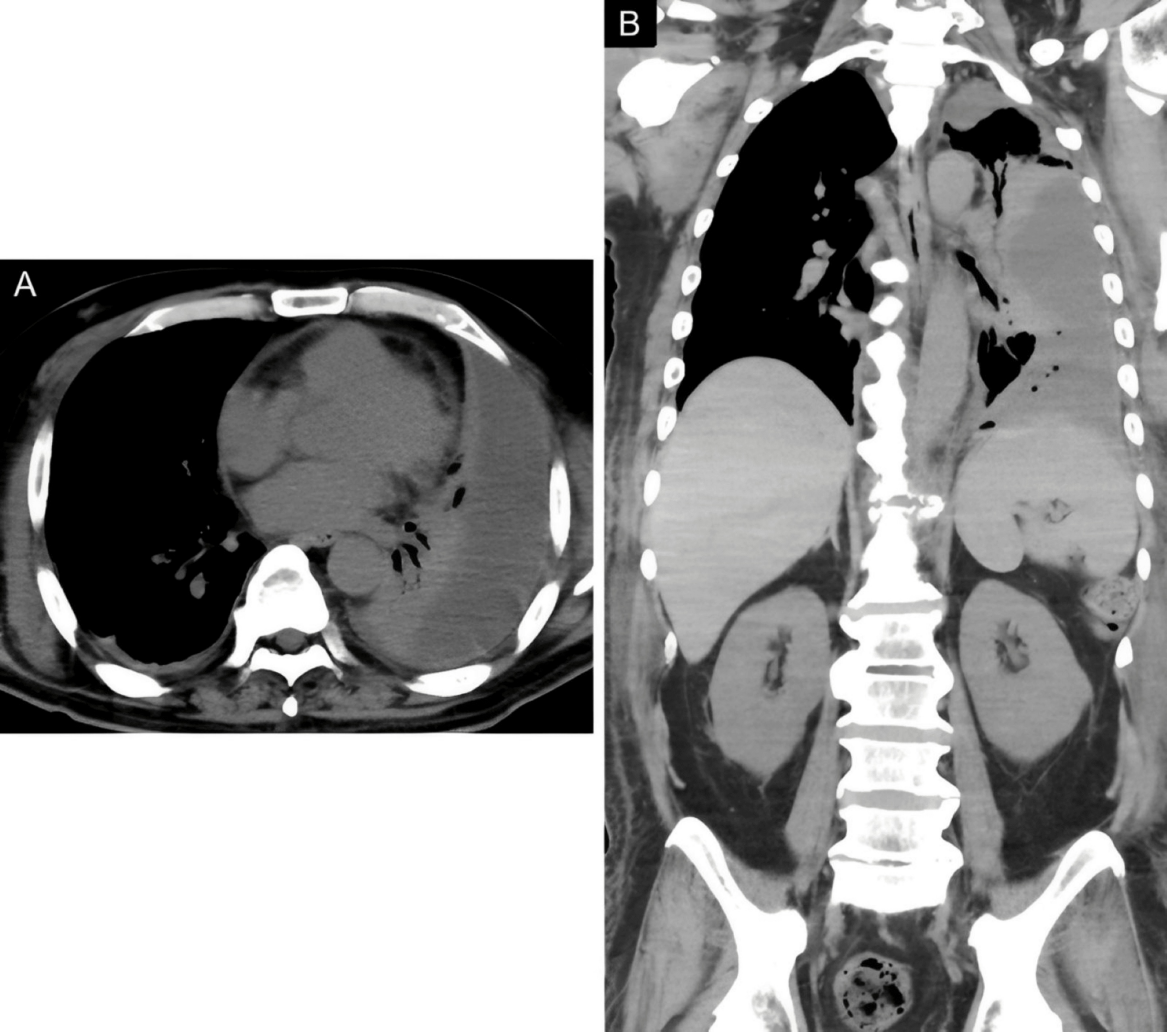
Non-contrast CT of the chest On day 21, left pleural effusion is depicted on CT, with values of 20 HU, associated with left lower lobe atelectasis (A, B). Note that small air bubbles are present in the fluid, which is consistent with pyothorax (B).

In addition, a hypodense fluid collection with gas locules within the left pleural space, with CT values of approximately 20 HU, was accompanied by compressive atelectasis of the left lower lobe (Figure 6). Moreover, intrapleural gas was noted, which was consistent with concurrent left-sided empyema.

Based on these findings, the patient was diagnosed with multifocal NSTI involving distant sites. Subsequently, left thoracic drainage, surgical irrigation, and left thigh debridement were performed. Ultimately, the patient underwent three debridement procedures, including additional treatment of the right forearm.

The patient responded to these treatments with gradual improvement in pain symptoms and inflammatory markers. Blood cultures obtained on admission and pus samples from debridement revealed *Staphylococcus aureus*, as shown in Table 2.

**Table 2:** Microbiological cultures and antimicrobial susceptibility of Staphylococcus aureus isolates Interpretations are based on CLSI M100-ED35 criteria. MIC, minimum inhibitory concentration; NA, not applicable; R, resistant; S, susceptible; TTP, time to positivity

Antibiotics	Blood culture (μg/mL)	Wound culture (μg/mL)	Pleural effusion (μg/mL)
Penicillins			
Ampicillin	0.5 (R)	0.5 (R)	0.5 (R)
Ampicillin/sulbactam	≤0.5 (S)	≤0.5 (S)	≤0.5 (S)
Piperacillin/tazobactam	1 (S)	1 (S)	1 (S)
Cephalosporins			
Cefazolin	≤0.5 (S)	≤0.5 (S)	≤0.5 (S)
Ceftriaxone	≤1 (S)	≤1 (S)	≤1 (S)
Cefmetazole	2 (S)	2 (S)	2 (S)
Carbapenems			
Meropenem	≤0.25 (S)	≤0.25 (S)	≤0.25 (S)
Macrolides and lincosamides			
Clarithromycin	≤0.5 (S)	≤0.5 (S)	≤0.5 (S)
Clindamycin	≤0.25 (S)	≤0.25 (S)	≤0.25 (S)
Quinolones			
Levofloxacin	≤0.5 (S)	≤0.5 (S)	≤0.5 (S)
Ciprofloxacin	≤0.25 (S)	≤0.25 (S)	≤0.25 (S)
Others			
Trimethoprim/sulfamethoxazole	≤5 (S)	≤5 (S)	≤5 (S)
Linezolid	1 (S)	1 (S)	1 (S)
Vancomycin	1 (S)	1 (S)	1 (S)
Minocycline	≤1 (S)	≤1 (S)	≤1 (S)
Fosfomycin	NA	NA	NA
Organism	S. aureus	S. aureus	S. aureus
Bacterial load/quantity	Positive (TTP: 24 hours)	Scant growth	Scant growth

**Figure 7:**
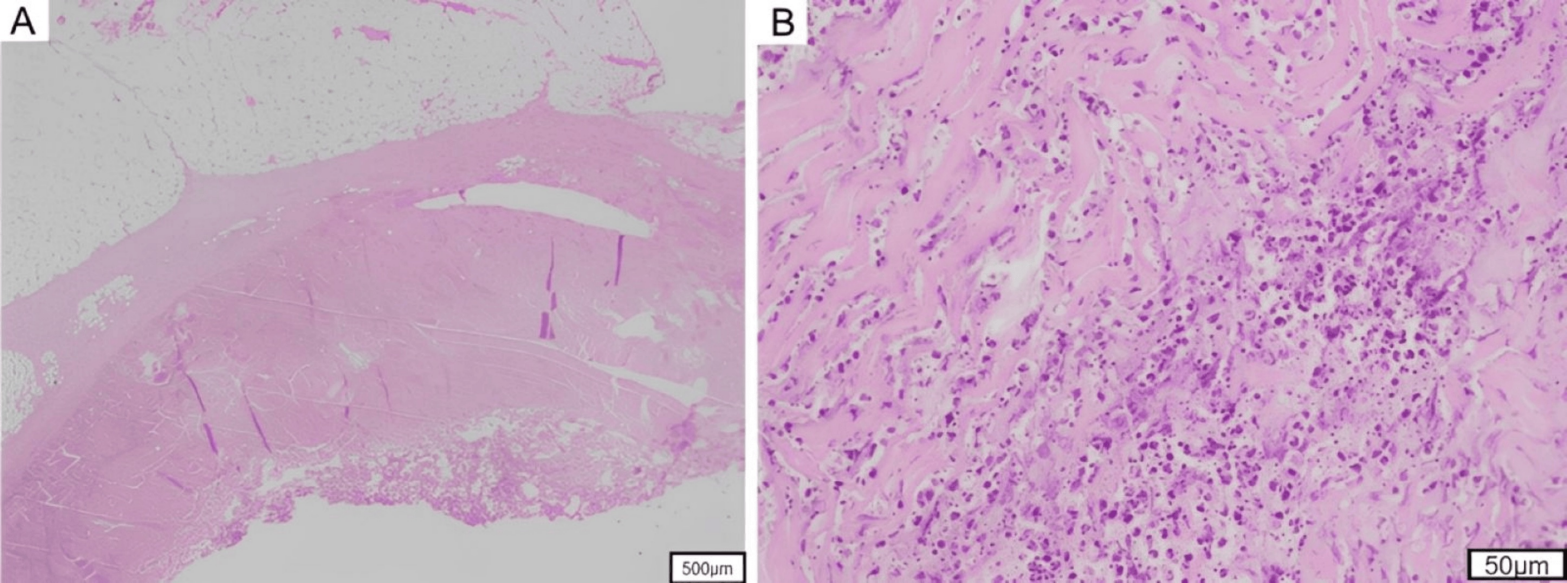
Histopathological section of debrided tissue Histopathological findings reveal marked infiltration of neutrophils and myonecrosis in the skeletal muscle layer, with minimal inflammatory cell infiltration in the subcutaneous adipose tissue (H&E staining). Magnification: (A) ×40; (B) ×100.

After continuing antibiotic therapy for approximately four weeks, the patient was discharged after a three-month inpatient course. Histopathological specimens from the left thigh debridement are shown in Figure 7A, 7B.

Subcutaneous adipose tissue exhibited minimal inflammation, whereas the fascia exhibited marked neutrophilic infiltration and fibrosis. The demarcation between the superficial and deep fasciae was indistinct because of fibrosis. Inflammatory cell infiltration extended into the skeletal muscle and was accompanied by myonecrosis.

## Discussion

This case represents a rare and life-threatening instance of NSTI involving multiple sites in the upper and lower limbs without apparent skin manifestations, complicated by concomitant empyema. Limb amputation was successfully avoided through prompt and accurate diagnosis and treatment, including multiple debridements, and the patient survived.

Regarding the onset patterns of NSTI, Stevens et al. reported two clinical presentations of invasive Group A *Streptococcus* (GAS) soft-tissue infection: those with a clearly identifiable portal of bacterial entry and those that occur de novo in deep soft tissues without any apparent trauma or preexisting skin lesions [4]. Approximately half of NSTIs caused by GAS are reported to arise without a discernible portal of entry, developing de novo in the deep soft tissue despite the absence of superficial skin or mucosal injury. In the present case, the causative organism was *S. aureus*, rather than GAS, and no obvious portal of entry was identified. Poor glycemic control due to diabetes possibly acted as a trigger, facilitating local proliferation of resident *S. aureus* and increasing its susceptibility to hematogenous dissemination. Hematogenous spread probably led to the establishment of infection in multiple deep-tissue sites. This case represents a rare pattern of NSTI onset and progression. Histopathological examination of the debrided tissues revealed extensive necrosis in the muscle fascia and subcutaneous tissue, with infiltration of inflammatory cells around the small vessels. These findings were consistent with hematogenous bacterial dissemination. Although the inflammation of the skin and subcutaneous fat was mild, marked inflammatory and necrotizing changes were observed in the deep muscle fascia and layers. This hypothesis was supported by microbiological and histopathological evidence.

Notably, Lee et al. compared the clinical characteristics and outcomes of patients with SMNSTI with those of patients with MoNSTIs and reported that multifocal cases were associated with more rapid progression and severe systemic manifestations. Underlying conditions, such as liver cirrhosis and end-stage renal disease, have been suggested as risk factors for SMNSTI [11]. In this case, the presence of diabetes and DKA possibly contributed to multifocal disease patterns. Therefore, when treating patients with underlying diseases, clinicians should consider the possibility of hematogenous dissemination and be aware that multiple lesions that are difficult to detect visually may be present.

However, in general practice, identifying the infection sites in NSTI cases complicated by bacteremia is not straightforward. Although severe pain is a characteristic symptom of NSTI, findings resembling cellulitis, such as erythema, swelling, and induration, are often minimal or absent. The affected area frequently lacks well-defined borders, and the pain is disproportionately severe compared to the appearance of the skin, making early diagnosis challenging. As the disease progresses, cutaneous manifestations, such as bullae, purpura, and necrosis, may appear; however, these are usually absent in the early stages. Infections can become rapidly systemic, leading to septic shock and DIC. Only 14% of NSTI patients are correctly diagnosed upon admission, reflecting a high risk of misdiagnosis [13,14]. A delayed diagnosis is directly linked to increased mortality and limb amputation rates. An accurate diagnosis is particularly challenging when severe pain is accompanied by minimal skin changes. Recent reports emphasize that imaging modalities, such as ultrasonography, CT, and MRI, should be appropriately combined to actively evaluate suspected infectious foci in NSTI, thereby facilitating earlier detection and intervention [13-20]. In the present case, the timely use of multiple imaging modalities undoubtedly contributed to the successful identification of the infection sites.

Generally, adequate source control measures in the treatment of NSTI require timely and appropriate surgical debridement. As demonstrated in the present case, accurate identification of infectious foci, especially when NSTI involves multiple sites rather than a single location, is essential for effective surgical intervention to remove and control the infection. This significantly influenced patient outcomes. Therefore, clinicians must meticulously ascertain the sites of infection, understand disease progression, identify causative pathogens, and consider patient comorbidities.

The causative microorganism is also an important factor that influences the prognosis of NSTIs. NSTIs are classified into two main types, based on the causative organism [4]. Type I infections are polymicrobial infections involving both aerobic and anaerobic bacteria and typically occur in elderly patients or those with underlying comorbidities. Type II infections are monomicrobial and most commonly caused by GAS, followed by methicillin-resistant *S. aureus*. Unlike type I infections, type II NSTIs can occur in individuals of any age, including those without underlying diseases. In the present case, *S. aureus* was isolated as the sole pathogen from cultures obtained from the wound, blood, and pleural abscess, providing an essential clue for understanding the disease pathology. This finding suggests that the multiple extremity lesions and truncal infection focus likely resulted from hematogenous dissemination. Furthermore, the identification of a commensal organism, such as *S. aureus*, as the causative pathogen, indicated that the patient was immunocompromised due to the underlying conditions. In addition, the relatively indolent clinical course of NSTI compared to that of NSTI caused by fulminant GAS may be attributable to differences in tissue invasiveness between these pathogens.

## Conclusions

This case involved type II SMNSTI caused by *S. aureus*, a condition typically associated with a poor prognosis, triggered by diabetes mellitus and DKA following septic shock. Despite the severity of the condition, the patient recovered after appropriate imaging-based evaluation and early therapeutic intervention. This case highlights that, although SMNSTI is an extremely rare entity, it demands high clinical vigilance to ensure prompt diagnosis and management. Further case studies are warranted to elucidate the pathogen-specific mechanisms underlying the onset and progression of soft tissue infections.
